# Efficacy, Safety, and Dose of Pafuramidine, a New Oral Drug for Treatment of First Stage Sleeping Sickness, in a Phase 2a Clinical Study and Phase 2b Randomized Clinical Studies

**DOI:** 10.1371/journal.pntd.0004362

**Published:** 2016-02-16

**Authors:** Christian Burri, Patrick D. Yeramian, James L. Allen, Ada Merolle, Kazadi Kyanza Serge, Alain Mpanya, Pascal Lutumba, Victor Kande Betu Ku Mesu, Constantin Miaka Mia Bilenge, Jean-Pierre Fina Lubaki, Alfred Mpoo Mpoto, Mark Thompson, Blaise Fungula Munungu, Francisco Manuel, Théophilo Josenando, Sonja C. Bernhard, Carol A. Olson, Johannes Blum, Richard R. Tidwell, Gabriele Pohlig

**Affiliations:** 1 Swiss Tropical and Public Health Institute, Basel, Switzerland; 2 University of Basel, Basel, Switzerland; 3 The Vaccine and Gene Therapy Institute of Florida, Port St. Lucie, Florida, United States of America; 4 Immtech Pharmaceuticals, Inc., Vernon Hills, Illinois, United States of America; 5 The QED group, Luanda, Angola; 6 Médecins sans Frontières Suisse, Genève, Suisse; 7 Lisumbi Health Centre, Kinshasa, Democratic Republic of the Congo; 8 Institut National de Recherche Biomédicale and Tropical Medicine Department, Kinshasa University, Kinshasa, Democratic Republic of the Congo; 9 Programme des Maladies Tropicales Négligées, Ministère de la Santé Publique Kinshasa, Democratic Republic of the Congo; 10 Ministry of Health, Kinshasa, Democratic Republic of the Congo; 11 Hôspital Evangélique de Vanga, Vanga, Democratic Republic of the Congo; 12 Federally Qualified Community Health Center, Elgin, Illinois, United States of America; 13 Instituto de Combate e de Controlo das Tripanossomíases, Luanda, Angola; 14 Infectious Diseases, Global Product Development, PPD, Rockville, Maryland, United States of America; 15 University of North Carolina, Department of Pathology and Laboratory Medicine, School of Medicine, Chapel Hill, North Carolina, United States of America; Hospital Infantil de Mexico Federico Gomez, UNITED STATES

## Abstract

**Background:**

Sleeping sickness (human African *trypanosomiasis* [HAT]) is caused by protozoan parasites and characterized by a chronic progressive course, which may last up to several years before death. We conducted two Phase 2 studies to determine the efficacy and safety of oral pafuramidine in African patients with first stage HAT.

**Methods:**

The Phase 2a study was an open-label, non-controlled, proof-of-concept study where 32 patients were treated with 100 mg of pafuramidine orally twice a day (BID) for 5 days at two *trypanosomiasis* reference centers (Angola and the Democratic Republic of the Congo [DRC]) between August 2001 and November 2004. The Phase 2b study compared pafuramidine in 41 patients versus standard pentamidine therapy in 40 patients. The Phase 2b study was open-label, parallel-group, controlled, randomized, and conducted at two sites in the DRC between April 2003 and February 2007. The Phase 2b study was then amended to add an open-label sequence (Phase 2b-2), where 30 patients received pafuramidine for 10 days. The primary efficacy endpoint was parasitologic cure at 24 hours (Phase 2a) or 3 months (Phase 2b) after treatment completion. The primary safety outcome was the rate of occurrence of World Health Organization Toxicity Scale Grade 3 or higher adverse events. All subjects provided written informed consent.

**Findings/Conclusion:**

Pafuramidine for the treatment of first stage HAT was comparable in efficacy to pentamidine after 10 days of dosing. The cure rates 3 months post-treatment were 79% in the 5-day pafuramidine, 100% in the 7-day pentamidine, and 93% in the 10-day pafuramidine groups. In Phase 2b, the percentage of patients with at least 1 treatment-emergent adverse event was notably higher after pentamidine treatment (93%) than pafuramidine treatment for 5 days (25%) and 10 days (57%). These results support continuation of the development program for pafuramidine into Phase 3.

## Author Summary

Sleeping sickness (human African *trypanosomiasis* [HAT]) is caused by parasites, and has a chronic progressive course that may last from several months to several years before death occurs. The present studies were done to assess the effectiveness and safety of oral pafuramidine versus intramuscular pentamidine (the standard treatment), in patients with first stage HAT. The results indicated that, several months after treatment, pafuramidine administered for 10 days was as effective as pentamidine administered for 7 days, and it had a better safety profile than pentamidine. With further study, pafuramidine could be a promising alternative for patients with first stage HAT. In addition, the design of the studies can be used a guide for future studies for identification and delivery of treatment to affected individuals in rural Africa.

## Introduction

Sleeping sickness (human African trypanosomiasis [HAT]) is a neglected tropical disease with limited treatment options that currently requires parenteral administration. It is caused by the protozoan parasites *Trypanosoma brucei (T*.*b*.*) gambiense* (the West African form of the disease) and *Trypanosoma brucei rhodesiense* (the East African form of the disease). *T*.*b*. *gambiense* is found in 24 countries in west and central Africa and currently accounts for over 98% of reported cases of sleeping sickness [1]. Sleeping sickness due to *T*.*b*. *gambiense* is characterized by a chronic progressive course, which may last from several months to several years before death occurs. The disease has two defined stages. The first stage is characterized by trypanosomes in the hemolymphatic system, which multiply in subcutaneous tissues, blood, and lymph. First stage symptoms entail bouts of fever, headaches, joint pains and itching. In the first stage, a person can be infected for months or even years without major signs or symptoms of the disease. When more evident symptoms emerge, the patient is often already in an advanced disease stage where the central nervous system is affected (second stage). The second (or neurological) stage begins once parasites penetrate the central nervous system, where their presence initiates deterioration in neurological function, including disruption of sleep/wake patterns that lend the name “sleeping sickness” to this stage of the disease [1].

After resurgence of the disease in the 1990s, the number of annual cases has subsequently dropped to less than 10,000 in recent years [2, 3]. Although the global incidence of HAT seems to be declining, several non-governmental organizations have cautioned that HAT still remains a “hidden epidemic” in regions impacted by civil war, such as the northeastern Democratic Republic of the Congo (DRC), southern Sudan, and the Central African Republic [4].

The socioeconomic burden on a household with an individual with HAT is high; between 1.5 and 10 months of household income can be lost, even when the diagnostics and antitrypanosomal drugs are provided free of charge. The extraordinary burden that HAT places on affected households and communities is often not very visible in national or regional health data because of its focal nature, and the disease often falls very heavily on a few locations [4].

The majority of current HAT research is focused on the second stage of the disease, which requires drugs that can cross the blood-brain barrier. The organo-arsenic compound melarsoprol has until very recently been the most widely used drug for treatment of second stage HAT, but because of its toxicity (encephalopathic syndrome, associated with 5–10% mortality), it is being progressively replaced by nifurtimox-eflornithine combination therapy [5]. The nifurtimox-eflornithine combination was placed on the World Health Organization (WHO) list of essential medicines and has been increasingly used [6, 7]. In addition, several new molecules have also shown promise for the treatment of second stage HAT. The most developmentally advanced of these compounds is fexinidazole, which has completed a Phase 1 clinical study and is now in Phase II/III evaluation [8].Two diamidine analogues [9, 10] and a benzoxaborole compound [11] have also exhibited promising activity (short *in vitro* time to kill and no cross-resistance) in animal models of second stage HAT.

An effective, safe drug for stage 1 HAT that can be easily administered in rural African settings is critically needed. There is no vaccine for *T*.*b*. *gambiense* HAT and only two drugs are approved for treatment of stage 1 disease: pentamidine (in the field only used for *T*.*b*. *gambiense*) and suramin (only for only for *T*.*b*. *rhodesiense*). In expatriate patients, pentamidine is currently the treatment of choice since is it generally well tolerated, whereas suramin can cause undesirable effects in the urinary tract in addition to allergic reactions [1]. Pentamidine is administered by the intramuscular route and has a reported treatment failure rate after a course of five injections of approximately 7% [12, 13, 14]. Despite this encouraging efficacy profile, treatment with pentamidine has limitations. It requires injection, which hampers its use in rural treatment facilities. Though adverse reactions are usually reversible and persistent manifestation of its most serious long-term consequence, diabetes, is rare, a high frequency of adverse events, including hypotension, nephrotoxic effects, leukopenia, and hypo- and hyperglycemia, has been noted [15, 16].

Pafuramidine (DB289) is the orally available dimethoxime prodrug of DB75 (furamidine), a novel diphenylfuran diamidine shown to be active *in vitro* against African trypanosomes and in animal models for trypanosomiasis [17, 18]. Pafuramidine is potentially a significant improvement over pentamidine, the drug currently used to treat first stage HAT. Its oral formulation greatly facilitates administration under challenging field conditions and makes pafuramidine readily available not only in sleeping sickness centers, but also public health facilities. Further, high doses of pafuramidine have been remarkably well tolerated in animal models of *trypanosomiasis* [19] and *Pneumocystis jiroveci* (a fungal infection of the lungs, formerly called *Pneumocystis carinii*, or PCP pneumonia) [20]. In addition, the prodrug pafuramidine and its active metabolite (DB75) have shown increased efficacy compared to pentamidine in animal models of *T*.*b*. *rhodesiense* infection [17, 19].

Prior to the initiation of the current Phase 2a study, pafuramidine had been successfully administered to healthy volunteers in both single-dose and multiple-dose studies, to evaluate the safety of pafuramidine and the pharmacokinetics (PK) of pafuramidine and DB75 [21]. Overall, multiple-dose treatment was well tolerated up to the maximum dose of 100 mg twice a day (BID), and was also well tolerated in the single-dose study up to 600 mg, although there was no substantially increased area under the plasma drug concentration versus time curve at doses above 100 mg.

The objectives of the present Phase 2 studies were to assess, for the first time, the efficacy, safety, and dosage of pafuramidine (Phase 2a), and to compare the efficacy, safety, and dosage of oral pafuramidine versus intramuscular pentamidine (Phases 2b and 2b-2) for treatment of first stage *T*.*b*. *gambiense* sleeping sickness. Pafuramidine may offer a potentially significant improvement over pentamidine, since it is orally administered and may be better tolerated.

## Methods

### Ethics Statement

All subjects provided written informed consent. This will certify that the Institutional Review Boards (IRBs) at the University of North Carolina at Chapel Hill, administered by the Office of Human Research Ethics, are organized and operate according to applicable laws and regulations governing research involving human subjects. These include, when applicable, statutes of the State of North Carolina and regulations of the Food and Drug Administration (21 CFR 50 and 56) and the Department of Health and Human Services [45 CFR 46 (the "Common Rule") and 45 CFR 164 (the Health Insurance Portability and Accountability Act, HIPPAA]. In addition, the IRBs conform, when applicable, to Good Clinical Practice (GCP) guidelines of the International Conference of Harmonization (ICH), to the extent these do not contradict DHHS and FDA regulations. The University of North Carolina at Chapel Hill holds a Federalwide Assurance, FWA 4801, approved by the federal Office for Human Research Protections (OHRP). These studies were approved by the following independent ethics committees: Ethikkommission beider Basel, EKBB, Comité de Ética Republica de Angola, and Comité Éthique République Démocratique du Congo. IRB# 01-PATH/LAB-308. International Protocol #289-C-006.

### Design

The Phase 2a study was a multi-center, multi-country, open-label, non-controlled, proof-of-concept study to assess the efficacy and safety of pafuramidine in 32 patients with first stage *T*.*b*. *gambiense* sleeping sickness. Patients were treated with 100 mg of pafuramidine orally BID for 5 days, and were hospitalized for a total of 12 days including a 6-day post-dose observation period. This study was conducted at two *trypanosomiasis* reference centers: one in Viana, Angola, and one in Maluku, Democratic Republic of the Congo (DRC) from 31 August 2001 (first patient enrolled) to 28 November 2004 (last patient follow-up completed). This study was approved by the following independent ethics committees: Ethikkommission beider Basel, EKBB, Comité de Ética Republica de Angola, and Comité Éthique République Démocratique du Congo.

The Phase 2b study was an open-label, parallel group, controlled, randomized trial to compare the efficacy and safety of pafuramidine with standard pentamidine treatment in 81 patients with first stage *T*.*b*. *gambiense* sleeping sickness. Patients were randomized (1:1) to either pafuramidine 100 mg BID administered orally for 5 days (n = 41) or pentamidine intramuscular injections (4 mg/kg QD) for 7 days (n = 40). This study was conducted at two sites: one *trypanosomiasis* reference center (Maluku, DRC) and one hospital (Vanga, DRC) from 01 April 2003 (first patient enrolled) to 08 February 2007 (last patient follow-up completed). This study also included a substudy/subset of patients enrolled at selected sites to assess the incidence and severity of laboratory anomalies (including aspartate aminotransferase [AST]/alanine aminotransferase [ALT] and glycemia) and incidence of electrocardiogram (ECG) anomalies.

The Phase 2b protocol was later amended to add an open-label study sequence (Phase 2b-2), in which an additional 30 patients were recruited at the same sites to confirm safety and efficacy of prolonged pafuramidine treatment. These patients received 100 mg of pafuramidine BID for 10 days with a total hospitalization time of 14 days.

The Phase 2b and 2b-2 studies were approved by the Ethikkommission beider Basel, EKBB, Committee on the Protection of the Rights of Human Patients, University of North Carolina Chapel Hill, and Comité Ethique Republique Democratique du Congo.

**Fig 1:**
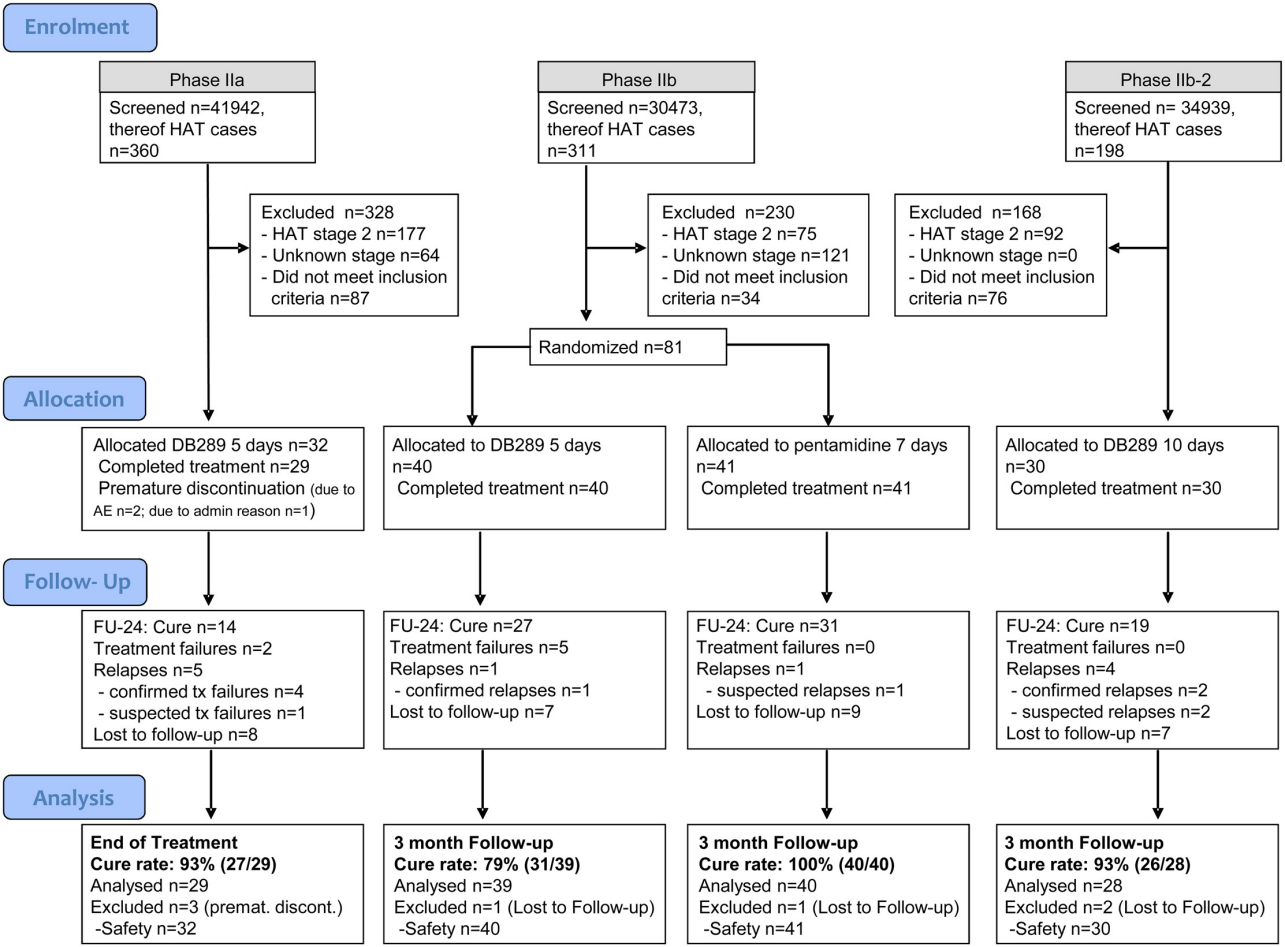
CONSORT flowchart for pafuramidine phase 2 studies.

In all studies, patients were stratified by site to ensure similar numbers of enrolled patients at each site. The flowchart for all the Phase 2 studies is depicted in Fig 1.

### Changes to Trial Design

All study amendments were approved by the ethical committees and IRBs previously noted. The Phase 2a amendment 1 included: 1) changed inclusion criterion for white blood cell (WBC) count from 20 to 5 cells/mm^-3^ to reduce the possibility of enrolling undetected late stage patients; 2) added a minimal weight of 40 kg to maintain consistency with Phase 1 trials; 3) added exclusion due to traumatic lumbar puncture to reduce the chance of incorrect staging of the disease at diagnosis; and 4) changed and refined the definition of treatment failures, based on the improved diagnostic tools to be used in the trial.

**Fig 2:**
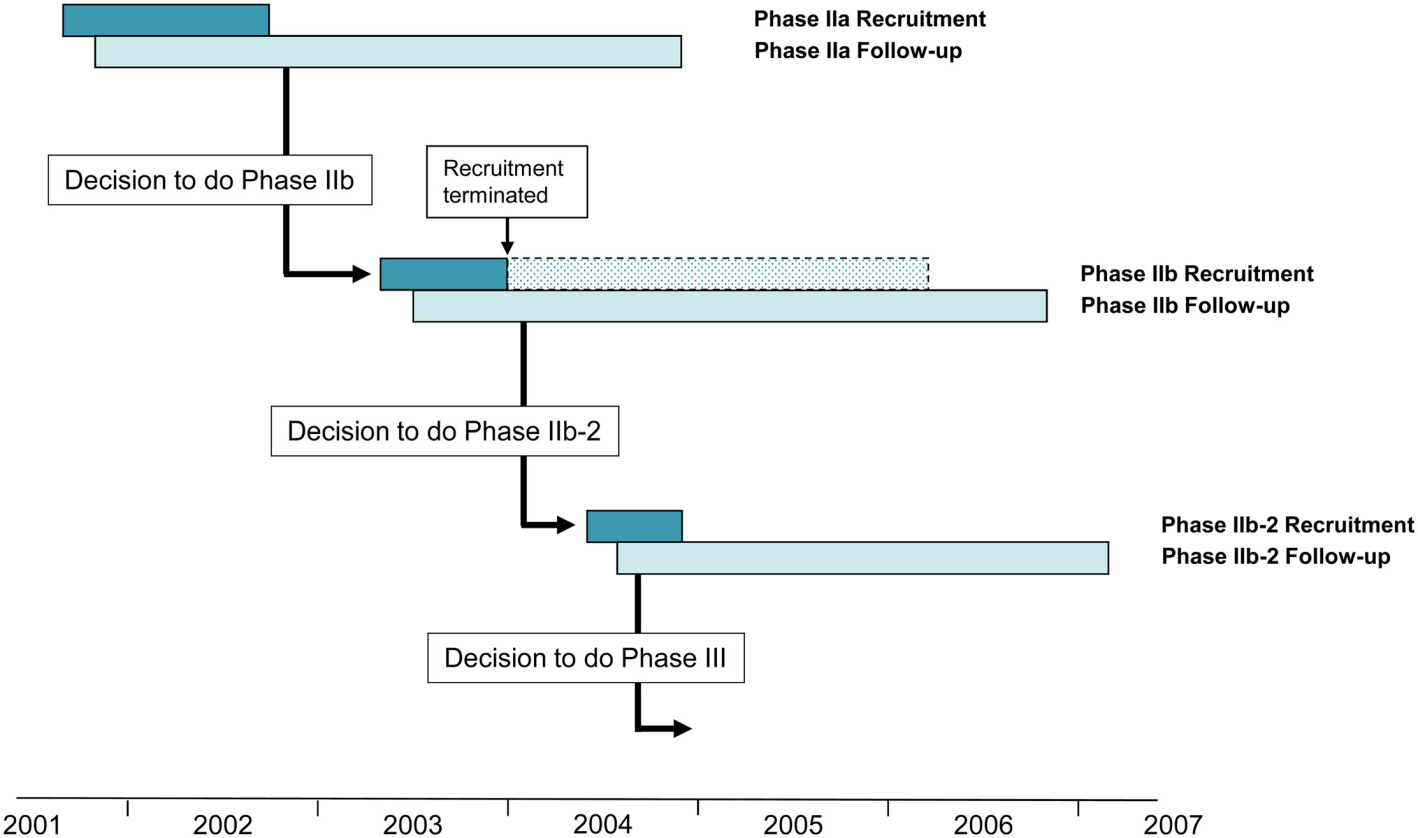
Schematic representation of the clinical development program.

Phase 2b amendment 1 added stricter rules for opening non-hospital-based sites for enrolment, by adding a “delay” of treating 40 patients. Phase 2b recruitment was subsequently terminated before opening accrual to rural sites and protocol modifications were implemented after five treatment failures were observed directly after treatment in the 5-day pafuramidine group in Phase 2a. After examination of the PK properties of pafuramidine, in particular, the lack of proportional conversion of DB289 to DB75 at therapeutic doses [22, 23], we decided against using a higher dose to improve efficacy for the modified protocol and instead increased pafuramidine treatment to 10 days at the same dose (100 mg BID) (Phase 2b-2) (Fig 2). Phase 2b amendment 3 added additional testing for parasites in lymph and circulating blood to reduce the potential for false-negative tests provides a chronological representation of the conduct of each study and illustrates the time points at which decisions were made, based on preliminary data, to proceed to the next study in the development program.

### Study Patients

Male and female subjects were eligible to participate if they had first stage *T*.*b*. *gambiense* sleeping sickness, documented by the presence of parasites in the blood or lymph and their absence in the cerebrospinal fluid (CSF), confirmed by <5 mm^-3^ WBCs detected in the CSF by microscopic examination. In the Phase 2a study, patients were 16 years or older, with a minimal weight of 45 kg, and in the Phase 2b study, patients were 15 to 50 years of age with a minimal weight of 35 kg. Women of childbearing potential were included if they were neither lactating nor pregnant, and were instructed to abstain from sexual intercourse from the day of consent until the end of the in-hospital observation period.

Key criteria for exclusion for all patients included 1) late stage *T*.*b*. *gambiense* infection; 2) active clinically relevant medical conditions that in the investigator’s opinion may have jeopardized patient safety or interfere with participation in the study (eg, significant liver diseases, chronic pulmonary diseases, significant cardiovascular diseases, diabetes, thyroid diseases, gout, infection including acquired immune deficiency syndrome, central nervous system trauma or seizure disorders); 3) traumatic lumbar puncture (red blood cells visible in CSF); 4) clinically significant abnormal laboratory values at screening; 5) score of less than 9 on the Glasgow Coma Scale; and 6) previous treatment for HAT.

### Interventions

Patients in all studies provided written informed consent. Patients in the Phase 2a study were screened either by mobile diagnostic teams or in the treatment centers. Patients with positive results at the mobile units were referred to the study sites for repeat testing. Patients in the Phase 2b and Phase 2b-2 studies were screened only in the treatment centers. Screening for *T*.*b*. *gambiense* was done using the card agglutination test for trypanosomes [24, 25]. All patients were tested for malaria and filaria in thick blood smears and, if indicated, malaria treatment was given before study enrolment; filariasis therapy was administered after study treatment.

All patients who had palpable lymph nodes underwent puncture at the screening or baseline visit. The aspirate was assessed microscopically for trypanosomes and if the result was negative, a blood sample was examined by hematocrit centrifugation and miniature anion exchange centrifugation technique. Lumbar puncture was performed in all positive cases detected by either method and the disease stage was determined by microscopic examination of CSF for trypanosomes and by WBC counts. Other screening and baseline evaluations included demographic and medical history, concomitant medications, vital signs, and physical examination including the Coma Scale. In all studies, baseline ECGs were performed and evaluated by a cardiologist to confirm patient inclusion.

Clinical supplies of pafuramidine were provided to the sites in bottles (50 gel caps of 100 mg) labelled to indicate study drug, strength, expiration date, protocol number, and other information according to local regulations. Pentamidine was provided locally as pentamidine isethionate for injection in single-dose vials at 200 mg per vial. Study drugs were stored at ambient temperature.

During the treatment periods in all studies, routine safety assessments included examinations for possible treatment-emergent adverse events, physical examinations, hematology tests (hemoglobin and leukocyte count), and chemistry profile (serum glucose, creatinine, AST, ALT, total bilirubin, urea, and C reactive protein).

In the Phase 2a study, patients were administered 100 mg of pafuramidine orally BID (morning and evening) for 5 days. In addition to the safety assessments previously mentioned, prothrombin time was also evaluated. For PK analyses, blood plasma levels were collected just before dosing and at several time points after the last dose. The 6-day post-dose observation period included additional ECGs after the last dose of study drug on Day 6; lymph node puncture and lumbar puncture on Day 7 (24 hours after last treatment, which is the primary endpoint); plasma samples for PK analyses; and a CSF sample for PK analysis on Day 7. Follow-up visits to evaluate treatment outcomes continued for 24 months, with lymph node punctures at Months 3, 6, 12, and 24 and lumbar punctures at Months 12 and 24.

In the Phase 2b study, patients received either pafuramidine 100 mg administered orally BID for 5 days or pentamidine intramuscular injections (4 mg/kg, once a day [QD]) for 7 days. Patients in both treatment groups stayed in the hospital for 7 days. In addition to the previously mentioned routine safety assessments, female patients in this study underwent pregnancy tests at baseline and end of treatment. A lymph node and lumbar puncture were also done at Day 7 (end of treatment). For patients in the substudy, blood samples for PK analyses were taken after the last dose on Day 6 and 7; ECGs were repeated on Days 1, 3, 7, and 9; and hematology and chemistry tests were repeated at Day 9. Follow-up visits to evaluate treatment outcomes continued for a period of 24 months, with lymph node, lumbar punctures, and a CSF sample at Month 3, 6, 12, and 24.

As part of Phase 2 amendment 2, patients received 100 mg of pafuramidine BID for 10 days with a total hospitalization time of 14 days. Study procedures in Phase 2b-2 were identical to Phase 2b, except the post-treatment procedures were done at Days 12 and 14. Follow-up visits to evaluate treatment outcomes also continued for a period of 24 months, with lymph node and lumbar punctures and a CSF sample as noted above.

#### Outcomes

The primary efficacy endpoint was the parasitological cure rate 24 hours after completion of treatment in Phase 2a (Day 7), and 3 months after completion of treatment in both Phase 2b studies. The primary determinants of parasitological cure were the absence of parasites in blood, lymph nodes, and CSF, as well as <5 mm^-3^ WBCs in the CSF 24 hours (Phase 2a) or 3 months (Phase 2b) after the last dose of pafuramidine. Parasitological cure/treatment failure was assessed after treatment by searching for the presence of parasites in blood and lymph by hematocrit centrifugation [26] and miniature anion exchange centrifugation technique [27].

A “relapse” was any recurrence of trypanosomes or surrogate signs during the follow-up period. Relapses included both confirmed and suspected relapses. For a confirmed relapse, trypanosomes were observed in blood, lymph, or CSF at any follow-up examination. For a suspected relapse, the WBC count increased to more than 50 cells mm^-3^ or the WBC count was 6 to 49 cells mm^-3^ and the patient had clear symptoms attributed to relapse (somnolence, long-lasting headache, and recurrent fever).

A “treatment failure” was a non-response to the study drug at the end of treatment. For a confirmed treatment failure, trypanosomes were found in blood, lymph, or CSF at post-treatment or any follow-up examination. A suspected treatment failure was reported if there was either 1) at least a fourfold increase in the serum titers of at least two of the applied tests compared to the post-treatment values or the preceding follow-up values, followed by a test repetition 1 month thereafter or 2) at least a fourfold increase in the CSF titers in the two applied tests compared to the post-treatment values or the preceding follow-up values, or an increase of WBC in the CSF above 20 mm^3^ followed by a test repetition 1 month thereafter in the absence of another likely diagnosis. Patients with treatment failures were treated (according to the national guidelines) with either pentamidine or melarsoprol. There was no discrimination between treatment failures and reinfections.

The primary outcome measure for the safety analysis in all studies was the rate of occurrence of WHO Grade 3 or higher treatment-emergent adverse events. The term “adverse event” could include any of the following events that developed or increased in severity during the course of the study: 1) any signs or symptoms whether thought to be related or unrelated to the condition under study; 2) any clinically significant laboratory abnormality; or 3) any abnormality detected during physical examination. Adverse events were graded by the investigator according to the WHO Toxicity Scale (1 = mild, 2 = moderate, 3 = severe, 4 = potentially life threatening). Adverse events were assessed at every study visit and were classified according to the terms found in the Medical Dictionary for Regulatory Activities (MedDRA).

A serious adverse event was defined as any event that suggested a significant hazard, contraindication, side effect, or precaution. A serious adverse event included any event that: 1) is fatal; 2) is life threatening; 3) is a persistent or significant disability/incapacity; 4) requires or prolongs in-patient hospitalization; 5) is a congenital anomaly/birth defect; or 6) is an important medical event, based upon appropriate medical judgment that may jeopardize the patient or may require medical or surgical intervention to prevent one of the other outcomes defined as serious.

#### Changes to outcomes

In the Phase 2 trial, every resurgence of parasite during the follow up and independent of the timing was considered as treatment failure. Based on consultation with the WHO for an upcoming Phase 3 trial, treatment failures (parasites detected at the 24-hour lumbar puncture performed after treatment) were distinguished from relapses (re-emergence of parasites at any time later during follow up). In the overall analysis both, treatment failures and relapses were considered failures.

### Sample Size

For the Phase 2a study, the sample size of 30 evaluable patients was considered to be sufficient to meet the primary objective because this size was comparable to a typical Phase 1 pilot study.

Based on a non-inferiority approach, calculation of the Phase 2b sample size assumed a 2% relapse rate in the pentamidine control group and a maximally accepted relapse rate of 10% in the pafuramidine treatment group. To achieve a power of 90% to refute the null hypothesis, in the event that the relapse rate of the new drug was equal to that of the standard (also 2%), 147 evaluable patients in each group were planned with a target enrolment of 175 patients in each group. A dropout rate of 15% was expected by the first follow-up examination at 3 months post-treatment.

The decision to continue the study in Phase 2b-2 with 30 evaluable patients was made based on assumption of a set rate of treatment failures (parasite-positive analyses 24 hours after treatment) and a binomial distribution for the number of relapses, and calculation of the probability to have N or more relapses. It was appropriate to make the nominal significance level smaller than 0.05 in order to keep the overall Type 1 error rate (the probability to incorrectly discard the treatment tested as not effective) at 0.05 [28]. For example, with a sample size of 30 and an assumed relapse rate of 1%, occurrence of 2 or more relapses had a 4% probability.

### Stopping Guidelines and Interim Analysis

No interim analysis was planned or conducted. There was no formal stopping rule for the Phase 2a and 2b trials. In case of accumulation of unexpected severe adverse events, the study director and the sponsor's medical director could have decided, after consultation with the advisory board chairperson, the investigator, and the country coordinator of the national HAT programs of Angola (ICCT), whether the trial needed to be stopped. In case of a major protocol violation by the investigator, the sponsor could have stopped the trial.

For Phase 2b-2, the decision to continue the trial was based on accepting (or assuming) a set rate of refractory cases (ie, a patient was parasite positive 24 hours after treatment) and a binomial distribution for the number of relapses. The probability to have N or more refractory cases in 30 patients can be seen in Table 1.

**Table 1:** The probability of refractory cases.

Total Number of Patients Treated	Set Rate of Refractory Cases Accepted	Observed Refractory Case (N)	Observed Rate of Refractory Cases	Probability of N or more cases
**Sequence 2**				
**30**	2%	3	10.0%	0.02
**30**	2%	2	6.6%	0.12
**30**	1%	1	3.3%	0.04

### Randomization

In all studies, patients were assigned a patient number in the order in which they enrolled, starting with Patient -001. In Phase 2a, there was no randomization since the study was open label. In Phase 2b, patients were randomized (1:1) in blocks of 10 in the order in which they were enrolled, stratified by clinical site, according to a randomization schedule prepared at Immtech International. Each study site was provided with series of individual envelopes each containing a card with the treatment assignment for 1 patient and a control number. After a patient signed the informed consent and inclusion/exclusion criteria were confirmed, the investigator opened the next envelope in the randomization list to obtain treatment assignment for that patient, then transferred the control number to the patient’s case report form.

Patients were identified on the case report forms by the patient number, initials, study center, and study identification numbers. The investigators kept a separate confidential enrolment log that matched identifying codes with the patients’ names and residences. The process was carefully monitored during the trial and envelopes were designed in a way that did not allow tampering or identification of the documentation inside.

### Blinding

There was no blinding in the studies due to the different routes of administration. Phase 2a and Phase 2b-2 were non-controlled studies.

### Statistical Methods

For the Phase 2a study, the parasitological cure rate was calculated as 100 minus the combined relapse and treatment failure rates at the specified time point. The denominator for computation of relapse rates was the number completing treatment.

The evaluable population used in the efficacy assessments reported for Phase 2b included patients who completed treatment with the assigned regimen of study medication and who either had a treatment failure or a relapse at any time prior to the scheduled time point of interest or underwent diagnostic procedures at the time point of interest or later. The last observation carried backwards was used for patients with missing data who had an assessment at a later visit, as long as the patient was not a relapse. For example, if a patient’s last visit was prior to 3 months (ie, the patient was lost to follow-up after the end-of-treatment visit), the patient was considered to be a relapse at 3 months.

For the Phase 2b and 2b-2 studies, the number and percentage of patients with parasitological cure at each evaluation was summarized by treatment group (pafuramidine for 5 days, pafuramidine for 10 days, and pentamidine for 7 days). The denominator included patients who had parasitologically confirmed infection with *T*.*b*. *gambiense* prior to treatment, completed the assigned regimen of study medication, and underwent diagnostic procedures at the end of treatment visit. All efficacy data were tabulated.

All patients who received treatment with the study drug were included in the analysis of safety and tolerability. In all cases, the primary outcome measure for safety analysis was the rate of occurrence of WHO Toxicity Scale Grade 3 (severe) or Grade 4 (potentially life-threatening) adverse events during the observation period. All safety data, including vital signs and adverse events, were tabulated.

All statistical analyses were performed with commercially available software (SAS version 9.0).

## Results

### Participant Flow

The Phase 2a study recruited patients from 31 August 2001 (first patient enrolled) to 28 November 2004 (last patient follow-up completed) and the Phase 2b study recruited patients from 01 April 2003 (first patient enrolled) to 08 February 2007 (last patient follow-up completed).

First stage HAT patients rarely present at a hospital or a treatment center. Therefore, intense screening activities were necessary to identify first stage patients. A total of 107,354 patients were screened to find 869 patients affected with HAT: 360 in Phase 2a, 311 in Phase 2b, and 198 in Phase 2b-2 (Fig 1). The exclusion rate was high (726 of 869 patients, 83.5%); primary reasons were stage 2 HAT, unknown disease stage, and inclusion criteria not met. Despite the high rate of screening exclusion, 32 patients were randomized and treated in Phase 2a, 81 patients in Phase 2b (40 to pafuramidine and 41 to pentamidine), and 30 patients in Phase 2b-2.

As shown in Table 2, study completion rates were high: 29 of 32 (90.6%) patients in Phase 2a, and 100% in both Phase 2b studies. In Phase 2a, 2 patients discontinued due to adverse events and 1 patient discontinued for administrative reasons. The follow-up attendance at Month 24 in all Phase 2b treatment groups was very good through the last assessment. A total of 83% (33 of 40) of patients treated with pafuramidine for 5 days, 78% (32 of 41) of patients treated with pentamidine, and 70% (21 of 30) patients treated with pafuramidine for 10 days underwent evaluation at the 24-month follow-up visit.

**Table 2:** Patient dispositions.

	Phase 2a Pafuramidine 100 mg BID (5 days) N = 32	Phase 2b N = 81		Phase 2b-2 Pafuramidine 100 mg BID (10 days) N = 30
		Pafuramidine 100 mg BID (5 days) N = 40	Pentamidine 4 mg/kg QD (7 days) N = 41	
**Randomized**	32	40	41	30
**Treated**	32	40	41	30
**Completed treatment**	29	40	41	30
**Discontinued treatment**	3	0	0	0
Withdrawn				
Adverse event	2			
Withdrawn				
Administrative	1			
**Evaluable time points**				
24 hours	29 (100%)	ND	ND	ND
3 months	28 (96%)	39 (98%)	40 (98%)	28 (93%)
6 months	27 (93%)	38 (95%)	39 (95%)	25 (83%)
12 months	24 (83%)	36 (90%)	36 (88%)	25 (83%)
24 months	21 (72%)	33 (83%)	32 (78%)	21 (70%)

BID = twice a day; QD = once daily; ND = not determined

### Baseline Data

Demographic variables (age, gender, weight, height, and body mass index) were summarized for all study patients (Table 3). Across studies, the treatment groups were similar with respect to age, distribution of men and women, height, weight, and body mass index.

**Table 3:** Baseline demographics.

	Phase 2a Pafuramidine 100mg BID 5 days (N = 32)	Phase 2b Pafuramidine 100 mg BID5 days (N = 40)	Phase 2b Pentamidine 4 mg/kg QD 7 days (N = 41)	Phase 2b-2 Pafuramidine 100 mg BID 10 days (N = 30)
**Age (years)**				
Mean (SD)	36.2 (11.03)	31.8 (10.47)	30.6 (10.73)	33.1 (11.99)
Median	38	32	31	32
Min, max	18, 63	15, 48	15, 50	15, 50
**Sex, n (%)**				
Female	18 (56)	25 (63)	22 (54)	21 (70)
Male	14 (44)	15 (37)	19 (46)	9 (30)
**Height (cm)**				
Mean (SD)	167.7 (7.73)	160.9 (7.32)	161.6 (8.30)	160.4 (8.27)
Median	168	160	160	160
Min, max	154, 182	150, 185	145, 177	142, 178
**Weight (kg)**				
Mean (SD)	56.5 (7.01)	48.7 (8.04)	48.5 (7.59)	47.8 (6.55)
Median	55	47	48	48
Min, max	46, 68	37, 69	34, 62	35, 59
**BMI (kg/cm**^**2**^**)**				
Mean (SD)	20.0 (1.35)	18.7 (2.50)	18.6 (2.60)	18.6 (2.12)
Median	20	18.1	18.3	18.6
Min, max	17, 22	14.7, 28.1	14.0, 26.0	14.9, 22.6

SD = standard deviation; BID = twice a day; QD = once daily; BMI = body mass index

Within the Phase 2b study, the treatment groups were also comparable in the time elapsed since symptoms were first observed, and the majority of patients in each treatment group were negative for malaria (range: 87% to 88%) and filaria (range: 83% to 100%), and did not have diarrhea at hospital entry (range: 98% to 100%).

### Numbers Analysed and Excluded

As shown in Fig 1, 29 of 32 patients (90.6%) in Phase 2a, 39 of 40 patients (97.5%) in the Phase 2b pafuramidine 5-day group, all 40 patients in the pentamidine group, and 28 of 30 (93.3%) of patients in the pafuramidine 10-day group were included in the efficacy analysis. There were only 7 patients excluded from the efficacy analysis: 3 in Phase 2a (due to premature discontinuation), 2 in Phase 2b (1 patient in each group lost to follow-up), and 2 patients in Phase 2b-2 (lost to follow-up). There were no protocol deviations.

#### Outcomes and estimations

As shown in Table 4, the parasitological cure rate 24 hours after the last treatment (primary endpoint) in the Phase 2a study was 93% (27 of 29 patients). All of the patients were negative for parasites in blood and CSF, while 2 patients remained positive for parasites in lymph nodes. The 93% cure rate (26 of 28 patients) was maintained at Month 3 and then declined to 67% (14 of 21 patients) at 24 months post-treatment. Five patients were considered relapses during the post-treatment follow-up period. This high number of relapses during the follow-up period of the Phase 2a study led to the termination of Phase 2b recruitment and revision of the protocol to a 10-day treatment period.

**Table 4:** Parasitological cure rates (primary and secondary efficacy endpoints).

	Phase 2a Pafuramidine 100 mg BID 5 days N = 32	Phase 2b		Phase 2b-2 Pafuramidine 100 mg BID 10 days N = 30
		Pafuramidine 100 mg BID 5 days N = 40	Pentamidine 4 mg/kg QD 7 days N = 41	
**24 hours**	93% (27/29)^a^	ND	ND	ND
**3 months**	93% (26/28)	79% (31/39)^a^	100% (40/40)^a^	93% (26/28)^a^
**6 months**	85% (23/27)	82% (31/38)	97% (38/39)	84% (21/25)
**12 months**	79% (19/24)	83% (30/36)	94% (34/36)	84% (21/25)
**24 months**	67% (14/21)	82% (27/33)	97% (31/32)	90% (19/21)

BID = twice a day; QD = once daily; ND = not determined

^a^ Primary efficacy endpoint

The Phase 2b cure rates at the primary endpoint (3 months after treatment) were comparable across treatment groups: 79% (31 of 39 patients) in the pafuramidine 5-day treatment group, 100% (40 of 40 patients) in the pentamidine 7-day group, and 93% in the 10-day pafuramidine extended treatment group (Table 4). Five treatment failures, all with persistent trypanosomes in the lymph nodes, were observed directly after treatment in the 5-day pafuramidine group

The rate of patients with parasitological cure during the follow-up period at 6, 12, and 24 months was slightly higher in the pentamidine group (97%, 94%, and 97%, respectively) compared to the pafuramidine 5-day group (82%, 83%, and 82%, respectively), but was comparable to the pafuramidine 10-day group (84%, 84%, and 90%, respectively) (Table 4). No treatment failures were observed directly after treatment in the pafuramidine 10-day group.

### Harms

All enrolled patients were included in the safety analysis. As shown in Table 5, in the Phase 2a study, the majority of adverse events were mild (Grade 1) or moderate (Grade 2) and the most commonly reported adverse events were headache (44%, 14 of 32 patients) and pyrexia (9%, 3 of 32 patients). There was only 1 patient with a severe (Grade 3) adverse event in the Phase 2a study (hypertension, unspecified, lasting for 1 day), which led to premature discontinuation of the study drug. One additional patient prematurely discontinued the study drug to an adverse event of moderate (Grade 2) pyrexia (the duration of the event is not available). There were no events higher than Grade 3.

**Table 5:** Treatment-emergent adverse events experienced by pafuramidin treated patients in the Phase 2a study.

MedDRA System, Organ, Class	Pafuramidine 100 mg BID 5 days (N = 32) n (%)
**Preferred Term**	
**Severe (Grade 3)**	
**Vascular disorders**	
Hypertension, unspecified	1 (3)
**Mild to moderate (Grade 1 or Grade 2)**	
**Nervous system disorders**	
Headache	14 (44)
**General disorders and administration site conditions**	
Pyrexia	3 (9)
**Gastrointestinal disorders**	
Abdominal pain upper	1 (3)
**Psychiatric disorders**	
Psychotic disorder, unspecified	1 (3)
**Skin and subcutaneous tissue disorders**	
Pruritus	1 (3)

BID = twice daily; MedDRA = Medical Dictionary for Regulatory Activities

No adverse event was considered possibly or probably related to the study drug and no serious adverse events were reported. There were minor increases in mean ALT, AST, and creatinine values, but these changes were not considered to be clinically significant; no patient experienced a ≥2-fold increase in ALT or AST.

As shown in Table 6, the overall rate of patients with treatment-emergent adverse events in the Phase 2b studies was higher among patients who received pentamidine (93%, 38 of 41 patients) than in patients who received pafuramidine for 5 days (25%, 10 of 40 patients) or 10 days (57%, 17 of 30 patients). The most commonly reported adverse events were ALT increased and AST increased, which were notably more prevalent in the pentamidine group than in either of the pafuramidine groups. Specifically, in the pentamidine group, 71% (29 of 41) of patients experienced increased ALT; of these, 16 had severe (Grade 3) elevations. In addition, a total of 85% (35 of 41) of pentamidine patients experienced increased AST. In the pafuramidine 5-day and 10-day groups, increased ALT occurred in 1 and 3 patients, respectively, and increased AST occurred in 4 and 5 patients, respectively. Elevations of liver enzymes in HAT patients were concurrent with treatment, were considered mild, resolved spontaneously (within 2–4 days), and were asymptomatic.

**Table 6:** Treatment-emergent adverse events experienced by ≥3 patients in any treatment group in the Phase 2b studies.

MedDR	Phase 2b		Phase 2b-2
A System Organ Class Preferred Term	Pafuramidine 100 mg BID 5 Days (N = 40) n (%)	Pentamidine 4 mg/kg QD 7 Days (N = 41) n (%)	Pafuramidine 100 mg BID 10 Days (N = 30) n (%)
**Total patients with at least 1 adverse event**	10 (25)	38 (93)	17 (57)
**General Disorders and Administration Site Conditions**			
Asthenia	1 (3)	1 (2)	5 (17)
Injection site pain	0	9 (22)	0
Injection site reaction	0	4 (10)	0
**Investigations**			
Alanine aminotransferase increased	1 (3)	29 (71)	3 (10)
Aspartate aminotransferase increased	4 (10)	35 (85)	5 (17)
**Metabolism and Nutrition Disorders**			
Anorexia	0	1 (2)	3 (10)

Other severe (Grade 3) adverse events included hypertension (1 patient in the pentamidine group) and headache (2 patients who received pentamidine and 1 patient who received pafuramidine for 5 days). All events of headache were considered to be related to trypanosomiasis and its treatment. No other Grade 3 or higher treatment-emergent adverse events were reported.

There were 2 serious adverse events in the Phase 2b studies. One patient who received pafuramidine for 5 days died 85 days after the last dose of the study drug. He was considered to have probable second stage disease with rapid progression, and death due to encephalopathic syndrome occurred during melarsoprol (rescue) treatment. One patient who received pentamidine was lost to follow-up after the 3-month evaluation and was subsequently reported to have died of causes not likely to be related to trypanosomiasis.

Overall, pafuramidine was well tolerated in all Phase 2 studies, and the rate of treatment-emergent adverse events was similar between the 5- and 10-day treatment groups. In the Phase 2b study, elevated ALT and AST values were more frequent in patients who received pentamidine 4 mg/kg QD for 7 days (71% and 85%, respectively) than in patients who received either pafuramidine 100 mg BID for 5 days (3% and 10%, respectively) or 10 days (10% and 17%, respectively).

Despite protocol-directed contraceptive measures, 2 pregnancies occurred during the treatment period, (1 in Phase 2a and 1 in Phase 2b-2). The courses of the pregnancies were normal and there were no abnormalities reported at birth. These children were repeatedly checked in the treatment center until the end of the study and reported to be in overall good health with normal development.

The ECGs from patients enrolled in these studies are included in a separately published study on cardiac alterations in HAT [29]. The results of the analyses suggested that a prolonged QTc interval, repolarization changes, and low voltage were significantly more frequent in first and second stage HAT patients compared with healthy individuals, which did not change during treatment with pafuramidine or pentamidine.

## Discussion

### Limitations

These were the first clinical studies in the field of trypanosomiasis conducted in the centers in Angola and the DRC with local teams that were previously inexperienced in clinical studies. However, they were fully compliant with Good Clinical Practice and regulatory standards.

### Interpretation

The results reported here demonstrate the efficacy of 5-day pafuramidine for treatment of *T*.*b gambiense* HAT with a 93% cure rate 24 hours post-treatment. Moreover, the parasitological cure rate 3 months after treatment was comparable in the pentamidine 7-day group (100%) and the 10-day pafuramidine treatment group (93%). The cure rate at 6, 12, and 24 months remained comparable between the pentamidine 7-day group (97%, 94%, and 97%, respectively) and the pafuramidine 10-day group (84%, 84%, and 90%, respectively). No treatment failures were observed directly after treatment in the pafuramidine 10-day group.

Pafuramidine was well-tolerated in a treatment regimen of 100 mg given orally BID for 10 days, and the toxicity appeared to be less than that observed for pentamidine 4 mg/kg QD given intramuscularly for 7 days. The efficacy (comparable to pentamidine) and safety (better tolerated than pentamidine) results obtained with the extended 10-day dosing regimen of pafuramidine support the continued clinical development of this drug.

### Generalizability

From the perspective of study design, it is noteworthy that the 3-month surrogate endpoint for efficacy used in the Phase 2b study effectively predicted the clinical outcomes determined at the 24-month evaluation. Given that this 3-month endpoint was implemented based on the progression of clinical efficacy observed in the Phase 2a study (with a primary endpoint at 24 hours post-treatment), the overall results represent one form of adaptive design for a series of studies within a clinical development program.

The conduct of the studies also established an organization and infrastructure that greatly facilitated the implementation of the subsequent Phase 3 study, eg, capacity building, improvement of laboratory infrastructure, investment in and deployment of high quality laboratory equipment, introduction of improved diagnostic methods, and improved experience in Independent Ethics Committee set-up and support.

As noted, the conduct of these studies required large-scale screening, which was supported by national HAT programs of Angola (ICCT) and DRC (PNLTHA). The success of the screening, in addition to the robust number of individuals who underwent screening, should contribute to improvement of HAT control in Angola and the DRC. The completion of the Phase 2 studies not only established the parameters for the design of a subsequent Phase 3 study, but also provided a model for future studies of HAT in these and similar populations, and a blueprint for the identification and delivery of treatment to affected individuals in rural Africa.

### Registration

Both clinical trials were registered in the International Clinical Trials Registry Platform at www.clinicaltrials.gov (Phase 2a study, NCT00802594; Phase 2b and 2b-2, NCT00803933).

## Supporting Information

S1 File CONSORT checklist. (DOC)
